# Gene and Protein Expression Is Altered by Ascorbate Availability in Murine Macrophages Cultured under Tumour-Like Conditions

**DOI:** 10.3390/antiox10030430

**Published:** 2021-03-11

**Authors:** Abel D. Ang, Margreet C. M. Vissers, Eleanor R. Burgess, Margaret J. Currie, Gabi U. Dachs

**Affiliations:** 1 Mackenzie Cancer Research Group, Department of Pathology & Biomedical Science, University of Otago Christchurch, Christchurch 8140, New Zealand; abel.ang@otago.ac.nz (A.D.A.); eleanor.burgess@otago.ac.nz (E.R.B.); margaret.currie@otago.ac.nz (M.J.C.); 2 Centre for Free Radical Research, Department of Pathology & Biomedical Science, University of Otago Christchurch, Christchurch 8140, New Zealand; margreet.vissers@otago.ac.nz

**Keywords:** ascorbate, tumour, tumour-associated macrophages, gene expression, microenvironment, hypoxia

## Abstract

Tumour-associated macrophages (TAMs) are ubiquitously present in tumours and commonly associated with poor prognosis. In immune cells, ascorbate affects epigenetic regulation, differentiation and phenotype via its co-factor activity for the 2-oxoglutarate dependent dioxygenase enzymes. Here, we determined the effect of ascorbate on TAM development in response to tumour microenvironmental cues. Naïve murine bone marrow monocytes were cultured with Lewis Lung Carcinoma conditioned media (LLCM) or macrophage colony-stimulating factor (MCSF) to encourage the development into tumour-associated macrophages. Cells were stimulated with hypoxia (1% O_2_), with or without ascorbate (500 µM) supplementation. Cells and media were harvested for gene, cell surface marker and protein analyses. LLCM supported bone marrow monocyte growth with >90% of cells staining CD11b^+^F4/80^+^, indicative of monocytes/macrophages. LLCM-grown cells showed increased expression of M2-like and TAM genes compared to MCSF-grown cells, which further increased with hypoxia. In LLCM-grown cells, ascorbate supplementation was associated with increased F4/80 cell surface expression, and altered gene expression and protein secretion. Our study shows that ascorbate modifies monocyte phenotype when grown under tumour microenvironmental conditions, but this was not clearly associated with either a pro- or anti-tumour phenotype, and reflects a complex and nuanced response of macrophages to ascorbate. Overall, ascorbate supplementation clearly has molecular consequences for TAMs, but functional and clinical consequences remain unknown.

## 1. Introduction

The immune response is crucial for tumour surveillance and eradication of established tumours [1]. However, cancer cells possess strategies to overcome immune surveillance, and once established, the ensuing tumour develops a microenvironment that not only suppresses the immune response but also promotes a wound healing inflammatory response that promotes tumour growth [2]. Tumour-associated macrophages (TAMs) play a significant role in supporting tumour growth and immune evasion [3]. They are present in most tumours and comprise of bone marrow-derived immature monocytes, circulating monocytes and resident macrophages [3]. Their prevalence is associated with poor prognosis in most tumours, with the exception of prostate and colon cancer [4]. Like most immune cells, macrophages are capable of phenotype switching from their unstimulated quiescent state (M0), into a spectrum of phenotypes that range between the classically described, and polar opposite, pro-inflammatory (M1) and anti-inflammatory (M2) state [5]. This phenotype switching or polarisation, largely depends on the environmental cues to which the immune cells are exposed. Several tumour-derived cues such as excessive lactate build-up [6,7,8] and presence of growth factors and cytokines [9,10,11] have been identified as polarising factors that lead to the development of an immunosuppressive and pro-tumour monocyte/macrophage phenotype. At present, there are no reports on the possible role that ascorbate may have on monocytes with regard to tumour immune response.

Immune cells are capable of taking up and retaining substantial amounts of ascorbate [12,13] via the sodium-vitamin C co-transporters (SVCTs) [14,15,16]. Freshly isolated peripheral lymphocytes, monocytes and neutrophils have an average of ~1, 3, and 1.5 mM intracellular ascorbate, respectively, and these values more than double when incubated with the upper limit of plasma ascorbate (100 µM) ex vivo [12]. These ascorbate levels have mostly been thought to provide an antioxidant defense mechanism owing to the natural ability to scavenge reactive oxygen species (ROS) [17]. In the context of TAM development however, the ROS scavenging activity of ascorbate is particularly pertinent, as it has been proposed that adequate intracellular ROS levels are required for macrophage differentiation into a more immunosuppressive TAM phenotype, and that ROS scavenging could inhibit this process [18,19,20].

Apart from being a classic ROS scavenger, more recently ascorbate has also been found to serve as a cofactor for numerous enzymes of the Fe^2+^ containing 2-oxoglutatrate dependent dioxygenase (2-OGDD) family [21]. This cofactor role of ascorbate is also attributed to its highly efficient physiological reduction of the active Fe^3+^ to Fe^2+^ [22,23], an oxidation state of iron that is required for 2-OGDD enzyme activity. Among the 2-OGDD proteins include the prolyl hydroxylases (PHDs), which control the activation of the hypoxia inducible factors (HIFs), and the Ten Eleven Translocase DNA demethylases (TETs) and Jumonji containing histone demethylases (KDMs), which have known roles in the epigenetic regulation of gene expression of immune cells [24]. Furthermore, it appears that millimolar ascorbate concentrations intracellularly are required for the optimal function of these enzymes and cells with high dependency on the epigenetic demethylases [23] [25,26,27,28]. This suggests an important role for ascorbate in immune cells beyond that of an antioxidant. 

In myeloid-specific HIF knockout experimental models, HIF expression was shown to be associated with TAM invasion [29], suppression of tumour cytotoxic T-lymphocyte responsiveness [30], increased expression of TAM Programmed death-Ligand-1 (PD-L1) [31] and tumour growth [29,30,32]. HIF activation is exacerbated when ascorbate availability is limited, and restricted access of ascorbate due to poor vascularity in solid tumours has been shown to be correlated with active HIF in tumour tissues [33,34,35]. Additionally, a proportion of patients with cancer are found to have low plasma ascorbate levels, some to the point of severe hypovitaminosis C [36,37,38,39,40,41]. Studies have also shown lower levels of circulating immune cell ascorbate in patients with cancer compared to healthy controls [37,42]. Ascorbate availability could also have an impact on hypoxia-mediated development and function of TAMs. It is uncertain however, if these lower levels of ascorbate in immune cells could affect the phenotype they acquire as they encounter the tumour microenvironment.

We hypothesise that ascorbate availability would influence gene expression and protein secretion of tumour-associated monocytes/macrophages. Here we analyse the impact of ascorbate on the phenotype of primary murine bone marrow-derived monocytes (BMDMs) grown in the presence of cancer cell conditioned media, without additional growth factors [6,43,44], and exposed to hypoxia (1% O_2_), a common feature in tumours known to polarise monocytes [45]. This system was used to mimic the milieu which monocytes would encounter as they extravasate from the circulation to infiltrate tumour tissue. We found differences between surface marker expression, gene expression and protein secretion of BMDMs grown with and without ascorbate.

## 2. Materials and Methods

### 2.1. Bone Marrow Derived Monocyte Isolation

Animal ethics approval was obtained from the University of Otago Animal Ethics Committee (AUP 18–144). For each independent experiment, 3–5 mice were used (total *n* = 20 mice). BMDMs were isolated from 8–16 weeks C57BL6 mice following established methods [46]. Briefly, mice were sacrificed by cervical dislocation, hind leg bones were then harvested, dipped in chlorhexidine (~1 s) and immersed for 5 min in growth media (Dulbecco’s Modified Eagle Medium (DMEM) (Life Technologies, Carlsbad, CA, USA)) supplemented with 10% foetal bovine serum (FBS) (Sigma Aldrich, Carlsbad, CA, USA), 1× non-essential amino acids (Life Technologies, Carlsbad, CA, USA), 8 mM Glutamax (Life Technologies, Carlsbad, CA, USA) and penicillin and streptomycin (50 units/mL) (Life Technologies, Carlsbad, CA, USA). The ends of the femur and tibia were then cut and marrow was flushed out with DMEM Growth Media using a 25 G needle. The marrow contents were then triturated vigorously to dissociate clumps of cells and passed through a 70 µM cell strainer (Corning, Corning, NY, USA) to yield the final bone marrow cell suspension in DMEM growth media.

To estimate ascorbate levels of nucleated whole bone marrow cells upon isolation, the final bone marrow cell suspension in one set of experiments was subjected to water lysis to remove erythrocytes. This involved the addition of Milli-Q water to final bone marrow cell suspension pellet, followed by gentle trituration and incubation for 15 s. One tenth volume of 10× phosphate buffered saline (PBS) (Life Technologies, Carlsbad, CA, USA) was then added to restore physiological osmolarity. Intact nucleated cells were then pelleted and reconstituted in serum-free DMEM.

### 2.2. Bone Marrow Derived Macrophage (BMDM) Culture

Isolated bone marrow cell suspension (20 mL from each mouse in DMEM) was mixed with Lewis Lung Carcinoma conditioned media (LLCM) (ratio of 3:2) or DMEM growth media (ratio of 3:2) with macrophage colony-stimulating factor (MCSF, 20 ng/mL) and cultured at a volume to surface area ratio of ~1 mL/4 cm^2^. This ratio of culture volume was selected to yield a consistent near confluent culture at day 7. Media was changed at day 2, 4 and 6, with day 6 having a growth media to LLCM ratio of 4:1. Fresh ascorbate (500 μM) was added at days 0, 2, 4 and 6 for the LLCM + Asc group. Non-adherent cells were washed off at day 2; cells did not adhere in the absence of MCSF or LLCM. Media and cells were harvested on day 7 for analysis. These cells were grown in incubators aerated with ambient air (~21% O_2_) and supplemented with 5% CO_2_. A separate set of cells from the same mouse were subjected to 1% O_2_ on day 6 using an H35 Hypoxystation (Don Whitley, Frederick, MD, USA), prior to harvest on day 7. These growth conditions will be referred to as 21% O_2_ and 1% O_2_, respectively. LLCM was prepared according to Colegio et al. [8], briefly, Lewis Lung Carcinoma cells (CRL-1642 from American Type Culture Collection, Manassas, VA, USA) were seeded at 2.4 × 10^5^ cells/cm^2^ and cultured with 0.36 mL/cm^2^ DMEM supplemented with 10% FBS and 1 mM sodium pyruvate (Life Technologies, Carlsbad, CA, USA) and final Glutamax of 8 mM for 48 h. LLCM was then harvested and centrifuged at 500× *g* for 5 min to remove any contaminating cells in the media and supernatants were stored at −80 °C.

### 2.3. Ascorbate Uptake Measurement

BMDMs were isolated and cultured for 7 days as described above. On day 7, cells were incubated with 50, 200 or 500 µM sodium ascorbate (Sigma Aldrich, St. Louis, MO, USA) and harvested at 30 min or 24 h to measure ascorbate content, or with 500 μM sodium ascorbate for 0, 2, 4, 6, 8 and 24 h. Wells were washed twice with PBS (Life Technologies, Carlsbad, CA, USA) and cells detached with two rounds of incubation in TrypLE (Life Technologies, Carlsbad, CA, USA) (200 µL each) and vigorous trituration. Cells were pelleted in microfuge tubes using a swing out rotor (500× *g* for 3 min at RT) and extracted with 0.54 M perchloric acid containing Diethylenetriamine penta-acetic acid (Sigma Aldrich, St. Louis, MO, USA) for ascorbate analysis [33]. Precipitated proteins were pelleted (10,000× *g* for 10 min at 4 °C) and ascorbate was measured in the supernatant using HPLC with electrochemical detection as previously described [34]. The concentration was assessed relative to standards ranging from 1.25 to 40 μM ascorbate made fresh for each analysis.

### 2.4. Immunostaining and Morphometric Analysis

Cells were stained for CD11b and F4/80 immunofluorescence in 24 well plates. At day 7, BMDMs were fixed with pre-chilled methanol (−20 °C) for 10 min at RT, washed three times with ice cold PBS and blocked with 2% bovine serum albumin (Life Technologies, Carlsbad, CA, USA) with 22.52 mg/mL glycine (Sigma Aldrich, St. Louis, MO, USA) for 45 min at RT. Primary antibodies and isotype controls were added in a 1% BSA PBS solution and incubated overnight at 4 °C (CD11b, Abcam (Cambridge, UK) #133357, 1:400; F4/80, Abcam #16911, 1:100). Wells were then washed three times with PBS 0.1% Tween (5 min per wash on orbital shaker) and incubated with fluorescent tagged secondary antibodies (1:500) for 1 h at room temperature (Anti-rabbit AF488, Abcam #150077; Anti-rat AF488, Abcam #150153). Wash step was repeated as described above and cells counterstained with DAPI (Sigma Aldrich, St. Louis, MO, USA), followed by three more washes. Finally, PBS was added to each well followed by imaging with an inverted microscope (Olympus IX81, Olympus, Tokyo, Japan).

### 2.5. Morphometric and Immunostaining Analysis

For morphometric analysis, cells were fixed using methanol, as described above. Phase contrast images from 4 random field of views (×20 objective) were captured for each culture condition and all complete cells within the images were assessed (an average of *n* = 1000 cells was measured per condition). The average cell aspect ratios of the 4 fields of view were then averaged to obtain the cell aspect ratio per condition per mouse. Using the Image J software (v 1.52P), the outlines of each cell was manually traced and automatically measured to obtain the length of the longest and shortest axis. The ratio of the longest to shortest axis was deemed as the cell aspect ratio.

For immunostaining analysis, fluorescent images from 4 random field of views (×30 objective) were captured for each culture condition and all complete cells within the images were assessed (an average of *n* = 1000 cells was measured per condition). The image capture conditions (exposure time) were kept consistent within each experiment in order to compare groups within an experiment. Using the Image J software (v 1.53C), the outlines of each cell was manually traced and automatically measured to determine the mean gray pixel value within each cell, as a measure of staining intensity. The average gray pixel value per cell of the 4 fields of view were then averaged to obtain the average cell gray pixel value (staining intensity) per condition per mouse.

### 2.6. Flow Cytometry

For flow cytometry analysis, BMDMs were grown in 60 mm dishes. The antibodies used for immunostaining were also used for flow cytometry. On day 7, dishes were rinsed thoroughly with PBS and incubated with 10 mM EDTA at 37 °C, followed by vigorous trituration to detach cells. This was repeated to recover remaining attached cells. Cells were collected, pelleted (500× *g* for 5 min) and reconstituted in 500 µL growth media on ice to block for 10 min. Subsequently, 100 µL aliquots were transferred to fresh microfuge tubes and incubated with primary antibodies (1:100) for 20 min on ice. Cells were then washed twice with PBS and reconstituted in 100 µL growth media containing fluorescently tagged secondary antibodies (1:100) and incubated for 20 min on ice. Cells were washed twice with PBS and reconstituted with 200 µL PBS for measurement on a flow cytometer (Beckman Coulter FC500 MPL, Beckman Coulter, Brea, CA, USA). 

### 2.7. Measuring BMDM Gene Expression

For gene expression analysis, BMDMs were grown in 60 mm dishes. At day 7, cells were washed twice with PBS and lysed with 500 µL Trizol (Life Technologies, Carlsbad, CA, USA) and transferred to a 1.5 mL microfuge tube. RNA was extracted as per manufacturer’s protocol. The final RNA pellet was reconstituted in 30 µL RNAse free water. RNA (1 µg) was reverse transcribed in a 50 µL reaction using the High Capacity cDNA Reverse Transcription kit (Applied Biosystems, Foster City, CA, USA) according to manufacturer’s protocol with 250 pmole random primers and 250 pmol Oligo(dT). Quantitative real time PCR was performed on cDNA using primers designed with the NCBI Blast engine for the following genes: *Arg-1*, *Cd206*, *Il-6*, *Il-10*, *Tgf-β1*, *Tnf-α*, *Vegfa*, *Ym1* (primer sequences are shown in Supplementary Table S1). Samples were amplified using BioTaq polymerase (Bioline, London, UK) in the presence of EVA green (Biotium, Fremont, CA, USA), 2.5 mM MgCl_2_, 0.2 mM dNTP, 0.2 µM primers and 40× diluted cDNA template. Samples were loaded onto a 384 well plate in triplicates and analysed using a Roche LightCycler^®^ 480 II. *β-actin* served as the endogenous reference gene and relative gene expression was calculated based on the method described by Pfaffl 2001 [47], with the assumption that the PCR efficiency for all genes was 2. The equation used is as follows:

*Relative gene expression* = 2 ^(**Ct Control** − **Ct Sample**) − (*Ct Control* − *Ct Sample*)^

Values in bold represent Ct values (cycle number that software calculates to be the earliest detectable point of replication) of gene of interest. Values in italics represent Ct values of the reference gene as an internal marker. All relative gene expression calculations were performed using MCSF grown bone marrow cells at 21% O_2_ as the ‘control’ and expressed as fold change.

### 2.8. Measuring Proteins by Antibody Array and ELISA

Culture media of three independent experiments were pooled together and assayed with an antibody array capable of detecting 308 proteins (Raybiotech L-308, Raybiotech, Peachtree Corners, GA, USA). At day 7, monocyte spent media was centrifuged at 1000× *g* for 5 min to remove cells prior to storage at −80 °C and subsequent assay. The amount of media sampled from each well was normalised based on total cell lysate protein content to ensure an even media volume per cell ratio. Protein measurements were obtained from total cell lysates of each well using a Bicinchoninic Acid Protein assay (Sigma Aldrich, St. Louis, MO, USA). Antibody arrays were imaged using UVITEC Alliance Q4 imaging system. Minimum threshold was determined from chemiluminescent signals derived from empty spots on the antibody array. Proteins that had relative luminescent signals below 250 units were deemed below threshold and were omitted from the final analysis.

The levels of a selected panel of cytokines (VEGF-A, TGF-β and IL-10) were quantified in the culture media of BMDMs at day 7 using commercial enzyme-linked immunosorbent assays (ELISA) kits (R&D Systems, Minneapolis, MN, USA) in three independent experiments with duplicate samples for each. Media was removed from BMDMs and centrifuged at 1000× *g* for 5 min to remove cells prior to storage at −80 °C and subsequent assay. To normalize ELISA data according to cell numbers, protein measurements were obtained from total cell lysates of each well as above.

### 2.9. Statistical Analysis

Each set of experiments was performed using cells obtained from an individual mouse, and all experiments had a minimum of *n* = 3–5 mice (i.e., *n* = 3–5 independent experiments, each with *n* = 2–4 technical replicates). Paired one and two tailed *t*-tests were used to assess differences in morphometric measures, surface markers, gene expression and ELISA in response to ascorbate supplementation. Statistical analysis was performed using GraphPad Prism version 7, with statistical significance set at *p* < 0.05.

## 3. Results

### 3.1. Cell Surface Markers of Isolated Bone Marrow Cells in Culture with LLCM and Hypoxia

**Figure 1:**
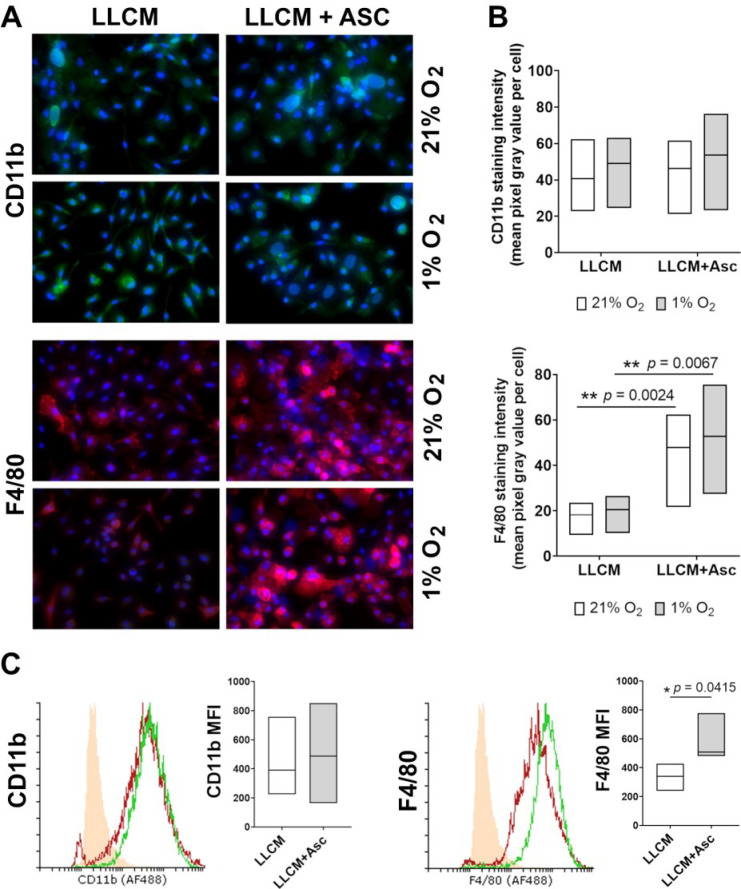
Characterization of cell surface markers of bone marrow cells grown in vitro. (**A**) Immunofluorescent staining of CD11b and F4/80 on bone marrow cells grown in LLCM with or without ascorbate (500 μM). Isolated bone marrow cells were grown for 7 days at ambient air (21% O_2_) or subjected to hypoxia (1% O_2_) on day 6 for ~18 h. Cells were then fixed and stained in situ for CD11b (green) and F4/80 (red); blue denotes DAPI staining for DNA. Microscope images (30×) are representative of results from one experiment (cells from one mouse). IgG control is shown in Appendix A, Figure A1. All growth conditions showed positive staining for CD11b and F4/80 in the majority of cells. (**B**) Densitometry analysis of CD11b and F4/80 immunostained cells represented by the mean gray pixel values per cell from 4 random fields of view per experiment (3 mice). CD11b staining did not show any statistical difference between groups. Cellular F4/80 staining was statistically more intense in the LLCM + Asc groups compared to LLCM group at the corresponding oxygen tensions. Two-tailed paired *t*-tests were performed, ** denotes *p* < 0.01. (**C**) Flow cytometric analysis of CD11b and F4/80 on bone marrow cells grown in LLCM with or without ascorbate at 21% O_2_. Isolated bone marrow cells were grown as in (**A**). Cells were then detached and stained for CD11b and F4/80 followed by flow cytometry analysis. Representative histograms from one mouse are shown. Solid orange histogram represents the negative control group (IgG isotype control), LLCM (red) and LLCM + Asc (green). All growth conditions showed a positive shift in CD11b and F4/80 stained cells (>90%) compared to IgG controls. Graphs show corresponding mean fluorescent intensity data from 3 individual mice, with each line representing paired outcome of one mouse. LLCM + Asc grown cells showed a significantly higher mean fluorescent intensity for F4/80 staining compared to LLCM only. Graphs shows bars with median ± min/max from 3 independent experiments. Two-tailed paired *t*-tests were performed, * denotes *p* < 0.05.

Bone marrow cells were analysed for myeloid (CD11b) and macrophage (F4/80) cell surface markers at day 7 to confirm lineage and cell purity of LLCM-grown bone marrow cells. Immunostaining revealed positive CD11b and F4/80 staining in most cells within randomly chosen field of views, in both LLCM and LLCM + Asc groups, at both 21% and 1% O_2_ culture conditions (Figure 1A). This was confirmed by flow cytometry, showing >90% positively stained CD11b and F4/80 cells grown from bone marrow with LLCM and LLCM + Asc supplementation, at 21% O_2_ tension (Figure 1C). We were unable to perform flow cytometry analysis on cells stimulated with 1% O_2_ because our method of staining did not involve cell fixation (due to antibody limitation), and it was not possible to stain and measure cells under constant hypoxic conditions. However, as the 1% O_2_ stimulation only took place on day 6 of the 7-day culture, and from the similar immunostaining observed in Figure 1A, we speculate that bone marrow cells had a high purity of myeloid cells of the monocyte/macrophage lineage under all culture conditions used. These cells will now be referred to as bone marrow derived macrophages (BMDMs) as F4/80 is reported to be generally low or undetectable in juvenile BMDMs and increase as they differentiate to macrophages [48].

Here, we also found cells grown in LLCM + Asc to have higher F4/80 staining intensity compared to LLCM only. Microscopic evaluation of immunostained cells showed higher average F4/80 staining per cell in the LLCM + Asc group for both oxygen tensions, but no change in CD11b staining (Figure 1B). Flow cytometric analysis supported this finding, with the LLCM + Asc BMDMs showing increased mean fluorescent intensity compared to the LLCM BMDMs (Figure 1C).

### 3.2. Ascorbate Uptake of LLCM-Grown Bone Marrow Derived Macrophages

**Figure 2:**
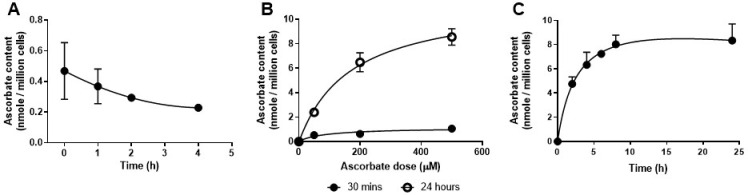
Ascorbate retention and uptake in LLCM-grown bone marrow derived macrophages. (**A**) Ascorbate levels were measured in freshly isolated mononuclear cells from bone marrow. Levels were detected upon isolation but reduced over subsequent hours. (**B**) Ascorbate uptake experiments were performed on LLCM-grown bone marrow derived macrophages at day 7. Dose response ascorbate uptake was measured at 30 min and 24 h post treatment. Minimal uptake was detected at 30 min across doses 50, 200 and 500 µM ascorbate. At 24 h there was a dose-dependent increase which appeared to plateau at 500 µM ascorbate. (**C**) Uptake of ascorbate over time was measured with treatment of 500 µM ascorbate. Ascorbate uptake began to plateau at about 8 h at ~ 8 nmol/million cells. Graphs represent data obtained from 3 independent experiments (mean ± SD). Note that, where not visible, error bars fall within the symbols.

Freshly isolated whole bone marrow cells were measured and found to have ascorbate levels of ~0.4 nmol/million cells (Figure 2A), similar to previous reports of whole bone marrow and peripheral leukocytes [49,50]. After 7 days of in vitro culture without ascorbate, LLCM grown BMDMs had no detectable levels of intracellular ascorbate, as shown in Figure 2B (cells with 0 μM ascorbate) and Figure 2C (measurement at time = 0 h). However, these cells were found to readily take up ascorbate as a function of concentration up to 500 µM, with minimal uptake at 30 min and increased uptake at 24 h (Figure 1B). At 30 min, ascorbate uptake of LLCM grown BMDMs was ~0.5 and 1.1 nmol/million cells at 50 and 500 µM, respectively, and this increased to ~2.4 and ~8.6 nmol/million cells at 24 h. At the upper concentration of 500 µM, maximal ascorbate uptake was estimated to occur at about 8 h (~8.0 nmol/million cells), and these levels were retained for up to 24 h (~8.3 nmol/million cells) (Figure 1C). Based on these results, we chose to use 500 µM as our ascorbate treatment concentration for the rest of the study as it was well tolerated by LLCM grown BMDMs at the ratio of cells/ascorbate used.

### 3.3. Morphology of LLCM Grown BMDMs and with Hypoxia Stimulation

**Figure 3:**
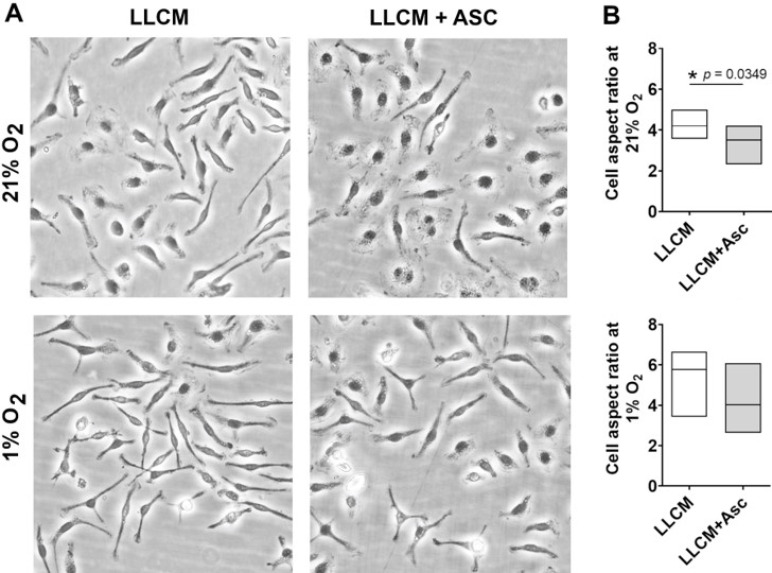
Morphology and morphometric analysis of LLCM-grown bone marrow derived macrophages in vitro. Isolated bone marrow cells were grown in cancer conditioned media (LLCM) or LLCM with 500 μM ascorbate (LLCM + Asc) for 7 days at ambient air (21% O_2_) or subjected to hypoxia (1% O_2_) on day 6 for ~18 h. (**A**) Images are representative using cells from one mouse (20×). Cell aspect ratio was determined by measuring the ratio of the longest cell axis to the shortest; higher ratios represents more spindled shaped cells. All conditions showed a mixed population of cells that ranged from spindled to spread out. (**B**) Cells grown with LLCM + Asc were less spindled overall compared to LLCM only, and statistical reduction in cell aspect ratio was observed at 21% O_2_. Graphs depict data obtained from 3 independent experiments (median ± min/max). Two-tailed paired *t*-tests were performed, * denotes *p* < 0.05.

Isolation and growth of BMDMs essentially relies on the adherent nature of monocytes to culture surfaces (plastic or glass) as well as continued survival and growth in the presence of specific growth factors such as MCSF. These conditions typically yield a purity of more than 90% after prolonged culture [46]. We found 40% LLCM supplemented media capable of supporting bone marrow cell adherence upon isolation and subsequent growth, while 10% and 20% LLCM supplementation was inadequate according to microscopic observations (data not shown). LLCM grown BMDMs at 21% O_2_ with or without ascorbate, resembled a mix of spindle shaped and spread-out cells (Figure 3A). These cells tolerated the switch from 21% O_2_ to 1% O_2_ (hypoxia) at day 6 for a period of ~18 h with no signs of cell attrition. However, after ~18 h of culture under hypoxia, LLCM grown BMDMs with or without ascorbate developed a more pronounced spindle shape with thinner and more elongated cell bodies. Ascorbate supplementation at 500 µM did not affect cell adherence, viability or growth. However, ascorbate supplemented LLCM grown cells were less spindled compared to LLCM-only cultured cells under both 21% and 1% O_2_ (Figure 3). Morphometric analysis supported visual observations although significant change was only found in cells grown under 21% O_2_ (Figure 3B).

### 3.4. Gene Expression of LLCM Grown BMDMs and with Hypoxia Stimulation

We performed quantitative PCR to measure changes in expression of genes encoding transcription factors, cytokines, enzymes and growth factors associated with polarisation status of macrophages. Changes in gene expression following isolation and expansion in LLCM, LLCM + Asc and different oxygen tensions were normalised to bone marrow cells grown in MCSF at 21% O_2_, representing a quiescent M2-like state [11].

**Figure 4:**
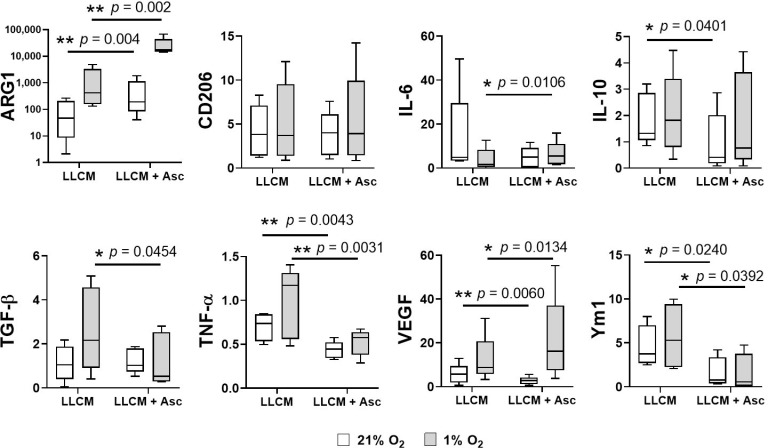
Gene expression analysis of LLCM-grown bone marrow derived macrophages with or without ascorbate. Isolated bone marrow cells were grown with MCSF, LLCM or LLCM + Asc for 7 days at ambient air (21% O_2_) or subjected to hypoxia on day 6 for ~18 h (1% O_2_). Cells were harvested for gene expression analysis by qPCR. Gene expression levels were calculated relative to bone marrow cells cultured with MCSF at 21% O_2_ and presented as fold change. Cells grown in LLCM showed altered gene expression levels compared to MCSF grown cells (**left** set of columns). Supplementation with ascorbate resulted in further significant gene expression changes (**right** set of columns). Oxygen tension also appears to alter expression of certain genes within each LLCM and LLCM + Asc cultured condition. Box and whiskers plot represent data from 5 independent experiments, box represents 25th to 75th percentile, whiskers represent min to max value and middle line represents median. Two-tailed paired *t*-tests were performed, * denotes *p* < 0.05 and ** denotes *p* < 0.01.

Compared to MCSF, LLCM grown BMDMs at both oxygen tensions showed increases in several classical M2-like and TAM-associated genes such as *Arg1, Cd206*, *Ym1* and *Vegfa* (left columns of Figure 4). Bone marrow cells grown in LLCM at 1% O_2_ showed a further increase in expression of TAM-associated genes *Arg1*, *Tgf-β*, *Tnf-a* and *Vegfa*, and a slight decrease in *Il-6*, compared to 21% O_2_ (comparing white and gray columns of the left columns).

Ascorbate supplementation in LLCM grown BMDMs further altered gene expression (right columns in Figure 4). At both oxygen tensions, there was a significant decrease in *Tnf-α* and *Ym1* expression in the presence of ascorbate, while *Arg1* showed a significant increase. Interestingly, ascorbate supplementation resulted in a significant decrease of *Vegfa* expression at 21% O_2_, but a significant increase at 1% O_2_. There were also other significant ascorbate-mediated gene expression changes that were observed only at specific oxygen tension; decrease in *Il-10* and *Tgf-β* at 21% O_2_ and 1% O_2,_ respectively, and increase in *Il-6* at 1% O_2_.

### 3.5. Protein Secretion of LLCM Grown BMDMs and with Hypoxia Stimulation

**Figure 5:**
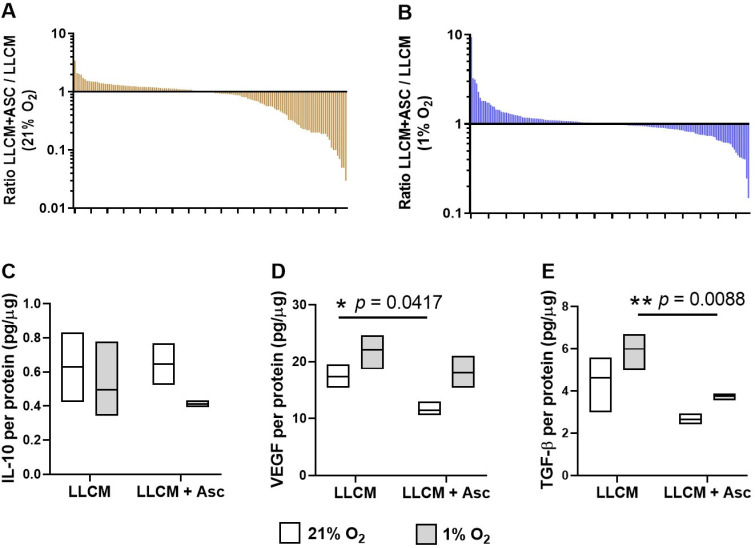
Effect of ascorbate on levels of protein analytes present in media of LLCM-grown bone marrow derived macrophages. Isolated bone marrow cells were grown with LLCM or LLCM + Asc for 7 days at ambient air (21% O_2_) or subjected to hypoxia on day 6 for ~18 h (1% O_2_). Media was refreshed on day 6 prior to harvest on day 7. Media from 3 independent experiments (3 mice) were pooled for a chemiluminescent protein array of 308 analytes. Graphs represent waterfall plots of the ratio of LLCM + Asc vs. LLCM of individual protein analytes present in media conditioned by BMDMs cultured at (**A**) 21% O_2_ and (**B**) 1% O_2_, respectively. At 21% O_2_, most of the observed change were ascorbate-mediated reduction in protein levels, while at 1% O_2_, there was a more balanced change in both directions. Selected secreted proteins were measured by ELISA, showing data for IL-10 (**C**), TGF-β (**D**) and VEGF-A (**E**). Box plots represent data from 3 independent experiments (median ± min/max). Paired *t*-tests were performed, * *p* < 0.05, ** *p* < 0.01.

To assess changes in protein secretion by LLCM grown BMDMs, we used a semi-quantitative 308 protein panel array. There were 134 and 158 proteins that were detectable at 21% and 1% O_2_, respectively. At 21% O_2_, LLCM grown BMDMs supplemented with ascorbate had 35 proteins that were decreased by more than 2-fold and 4 proteins that increased by more than 2-fold (Figure 5A). At 1% O_2_, LLCM grown BMDMs supplemented with ascorbate had 8 proteins that were decreased by more than 2-fold and 6 proteins that increased by more than 2-fold (Figure 5B). This ascorbate dependent change in protein profile also differed between cells grown at 21% and 1% O_2,_ with respect to both protein species and overall direction of change (Figure 5A,B). To provide an overview of all changes, Table S2 shows the full list of proteins measured.

The antibody array data suggested ascorbate-mediated changes in abundance of several proteins with known pro- or anti-tumour roles. The top proteins altered in abundance following ascorbate supplementation are shown in Table 1. Among the proteins with the highest increase with added ascorbate in ambient air were VE-cadherin (3.5 fold) and under hypoxia, TNF-related apoptosis-inducing ligand (TRAIL) (9.4 fold) and insulin-like growth factor binding protein 7 (IGFBP-7) (3.3 fold). The secreted proteins most decreased by ascorbate included FGF R3 (−33 fold), MCP-1 (−20 fold) and bFGF (−2.5 fold).

Protein levels of selected secreted cytokines were measured by ELISA (Figure 5C–E). Ascorbate supplementation significantly decreased secretion of TGF-β under hypoxia (Figure 5D, *p* = 0.009) and VEGF-A (Figure 5E, *p* = 0.042 for 21% O_2_, *p* = 0.055 for 1% O_2_), whereas no significant changes were seen for IL-10 (Figure 5C).

## 4. Discussion

Monocytes and macrophages are highly plastic cells capable of phenotypic change in response to environmental cues. While this is crucial for coordinating an inflammatory response and maintaining homeostasis, this plasticity can be subverted by tumours to push monocytes and macrophages to an M2 wound healing-like phenotype, which suppresses the immune response and promotes tumour growth and metastasis [3]. The findings of this paper show that ascorbate alters monocyte gene expression and protein levels in response to growth and differentiation with tumour microenvironment cues, which could impact the function of these cells.

For this study, we used bone marrow as a source of monocytes and have demonstrated: (1) that monocytes grown in LLCM-conditioned media showed increased expression of M2-like and TAM genes compared to cells grown in MCSF and provide a relevant system to study tumour driven differentiation of monocytes; (2) that the expression of M2-like and TAM genes was further increased under hypoxia; (3) that ascorbate was a requirement for increased cell surface expression of F4/80 in LLCM grown BMDMs, a maturation and differentiation macrophage marker; 4) that ascorbate modified LLCM grown BMDM gene expression for proteins associated with angiogenesis, epithelial to mesenchymal transition, immunosuppression and M2 markers, in both normoxic and hypoxic conditions. These are all novel findings that will inform our understanding of the impact of variations in the tumour microenvironment on cancer growth.

We detected low intracellular levels of ascorbate in whole bone marrow mononuclear cells upon isolation (~0.4 nmol/million cells), in agreement with a previous report showing similar ascorbate levels in whole bone marrow leukocytes (~0.3 nmol/million cells) [49]. Higher ascorbate levels were however reported for haematopoietic progenitor cells of increasing stemness (~1.2–2.5 nmol/million cells) [49]. LLCM grown BMDMs were found to readily take up ascorbate and achieve saturation at ~8 nmol/million cells with 500 μM ascorbate, in agreement with a previous report in human peripheral monocytes saturating at ~3 nmol/million cells when supplemented with 100 µM ascorbate [12].

We cultured primary bone marrow monocytes solely in the presence of LLCM (40% *v*/*v*) without additional growth factors that are essential for survival, growth and differentiation of naïve monocytes [6,43,44]. This is vital for the in vitro study of TAM development and phenotype, as the common practice of monocyte expansion with purified growth factors prior to tumour-related stimulation results in pre-differentiated monocytes [11], thus skewing the in vitro development of TAMs from primary monocytes. However, it is important to note that this model system is still a simplified approximation of the tumour microenvironmental cues which involve complex interplays between various cell types, extracellular matrices and physical stressors. LLCM supplementation was capable of supporting monocyte/macrophage expansion with >90% cell purity. In addition, ascorbate markedly increased cell surface expression of F4/80 in bone marrow cells. The human analogue of F4/80, Adhesion G Protein-Coupled Receptor E1 (ADGRE1), is similarly expressed in circulating monocytes and tissue macrophages [51]. F4/80 is an adhesion G-protein coupled receptor with poorly understood function. Evidence suggests that it is involved in natural killer cell–macrophage cellular contacts necessary for mediating a robust interferon-γ response, but also induction of peripheral tolerance [52]. Therefore, the ascorbate induced increase in F4/80 monocyte cell surface expression could have a pro- or anti-tumour immune related effect.

Growth in cancer-conditioned medium increased expression of M2-like and TAM associated genes compared to MCSF, supporting the concept of cancer cells polarising monocytes towards an M2-like pro-tumour state. Among these were genes that promote angiogenesis (*Vegfa*), epithelial to mesenchymal transition (*Tgf-β*), immunosuppression (*Tgf-β*, *Il-10* and *Arg1*), and classical M2 markers (*Cd206* and *Ym1*). Hypoxia, a known regulator of tumour immune response [45], served as an additional stimulus to further increase expression of *Arg1*, *Tgf-β*, *Ym1* and *Vegf*. Ascorbate treatment, on the other hand, lowered expression of *Ym1*, *Il-10* and *Vegfa* but increased *Arg1* in ambient air. Under hypoxia, ascorbate lowered expression of *Ym1*, *Tgf-β* but increased *Arg1* and *Vegfa*. Interestingly, ascorbate also affected the expression of classically pro-inflammatory M1 genes by increasing *Il-6* expression but decreasing *Tnf-α*.

Ascorbate-mediated gene expression changes were largely reflected in secretome content as measured by antibody array and ELISA. However, it is well documented that gene expression and protein abundance are not always congruent [53,54]. Some discrepancies could be attributed to protein consumption by the BMDMs themselves and the protein content of LLCM, as qPCR data merely reflects the cells’ potential to express proteins of interest. In LLCM grown BMDMs cultured at 21% O_2_, there was a general trend of ascorbate-mediated reduction in both gene expression and secretion of pro-tumour proteins, such as the immunosuppressive and anti-inflammatory cytokines IL-10 [55] (qPCR and antibody array) and TGF-β (qPCR and ELISA), as well as the pro-angiogenic factor VEGF-A [55] (qPCR, antibody array and ELISA). Under hypoxia, this trend was less apparent, with only TGF-β, an inducer regulatory T cell development and promoter of epithelial to mesenchymal transition, showing an ascorbate-mediated reduction [55] (qPCR, antibody array and ELISA). Under hypoxia, as opposed to ambient air conditions, VEGF-A showed an ascorbate-associated increase in expression and secretion (qPCR and antibody array). This altered VEGF expression and secretion could be due to the involvement of IL-10 in maintaining macrophage VEGF production, particularly under hypoxic conditions, as has been shown previously [56,57]. Overall, ascorbate supplementation was shown to modify the expression and secretion of various key monocyte/macrophage proteins in response to growth in cancer cell conditioned media. Our data suggest that ascorbate supplementation skewed monocytes towards a lesser pro-tumour-like phenotype under normoxic conditions but this was less apparent with hypoxic stimulation.

Of the secreted protein, TRAIL was found to be highest among ascorbate-mediated proteins. TRAIL is secreted by monocytes and is classically known as an anti-tumour protein as it selectively promotes apoptosis following binding to, and ligation of, Death Receptors and Decoy Receptors on the surface of several tumour cell lines [58,59]. However, TRAIL signaling is more complex than originally thought, being involved in regulating immune and inflammatory cells as well [60], and promoting a tumour-supportive immune microenvironment [61]. Among other top ascorbate-mediated protein increases were extracellular matrix remodeling proteins such as MMP-2 and TIMP-1 which are associated with tumour metastasis and poor prognosis [62]. On the other hand, ascorbate treatment was also associated with increase in IGFBP7, a known tumour suppressor [63,64,65] that is thought to compete with insulin-like growth factor binding [66] or cause oncogene-induced senescence [67], and has been recently shown to be produced by macrophages [68]. Other notable candidates reduced by ascorbate are bFGF, considered a pro-tumour protein involved in tumour angiogenesis [69], and MCP-1, involved in recruitment of myeloid derived suppressor cells [70,71] but also found to be secreted by myeloid derived suppressor cells and responsible for tumour survival and metastasis [72]. These changes, though potentially important, need to be validated by quantitative methods of analysis.

Apart from changes in secreted proteins, several membrane proteins were also present. Shedding, a commonly occurring mechanism for controlling levels of membrane proteins, is primarily mediated by A Disintegrin and Metalloproteinases (ADAMs) [73], which are present on the surface of tumour associated monocytes [74]. Among the membrane proteins detected at modified levels were TYRO3, a member of the receptor tyrosine kinase family [75] involved in macrophage dependent efferocytosis and acquisition of pro-tumour phenotype [76], LRP-6, a low-density lipoprotein-related co-receptor that signals via Wnt/β-catenin [77] known to influence macrophage differentiation, migration and function [78]. Taken together, this could suggest a shift in monocyte function, but further validation is required.

It is important to note here that the antibody array is an exploratory method and would require more rigorous quantitative measurements to validate current findings. However, as with the qPCR data, the antibody array also yielded outcomes that could support both pro and anti-tumour affects following ascorbate supplementation to LLCM grown BMDMs. Although our data point to a potential effect of ascorbate on macrophage function in the tumour microenvironment, a more in-depth analysis of the functional consequences of ascorbate availability was beyond the scope of this study. This would require specific gain-of-function or loss-of-function studies using gene-editing that is challenging in primary cells. Adaptation of our findings to a model system using immortalized cell lines could be informative in this regard.

There is interest in the role of intracellular ascorbate and its effect on the tumour immune response. In vivo studies utilised ascorbate deprived or supplemented mice, followed by observations of tumour immunological response, without any direct correlation between immune cell ascorbate levels and phenotypic change [79,80]. Two in vitro studies have associated higher intracellular ascorbate levels of freshly isolated human CD8+ T cells and NK cells with increased cytotoxicity towards leukaemic cells [79,81]. However, there have been no studies investigating the effect of intracellular ascorbate on the immune cell phenotype under the burden of cancer. Our study provides direct evidence that intracellular ascorbate levels affect gene and protein expression in monocytes grown under in vitro tumour microenvironmental conditions. This alludes to a possible role of ascorbate in the monocyte tumour response. However, further studies are required to determine if these changes would have functional consequences.

## 5. Conclusions

The immune response plays a crucial role in the surveillance and elimination of developing tumours. However, the immune response is often circumvented and subverted to promote tumour growth and progression instead, owing to the immune phenotype altering cues present within the tumour microenvironment. Here, we have shown that ascorbate-loaded murine monocytes respond differently to ascorbate-depleted monocytes, when grown under conditions that simulate the tumour microenvironment in vitro. Ascorbate-loaded monocytes demonstrated changes in cell surface marker and gene expression, as well as protein secretion, compared ascorbate depleted monocytes, but these were neither clearly pro- or anti-tumour associated. Our data suggests that monocyte ascorbate levels could modify their phenotype and potentially affect their function within the tumour, but further studies, including the use of selective gene editing, could be informative in determining the specific pro- or anti-tumour effects. On balance, our data demonstrate a complex and nuanced response of monocytes to ascorbate.

## Figures and Tables

**Table 1:** Top ten secreted proteins differentially regulated by ascorbate in media conditioned by BMDM cultured at 21% and 1% O_2_.

Ratio LLCM + Asc/LLCM	21% O_2_	1% O_2_
Top 5 increased	VE-cadherin (3.5), MMP-2 (2.1), LRP-6 (2.0), IL-1 R6 (2.0), Neurturin (1.7)	TRAIL (9.4), IGFBP-7 (3.3), TYRO3 (3.2), MMP-2 (2.8), TIMP-1 (2.3)
Top 5 decreased	FGF R3 (−33), Granzyme D (−20), MCP-1 (−20), TLR4 (−14), IL-5 Rα (−13)	CCR4 (−6.7), IL-2 (−4.2), bFGF (−2.5), Decorin (−2.4), IL-6 (−2.3)

Values in brackets represent fold change of secreted proteins of LLCM + Asc/LLCM.

**Figure A1:**
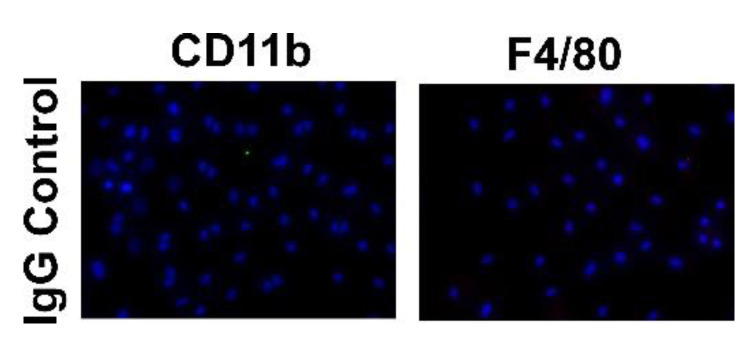
IgG control immunostaining of bone marrow cells. Isolated bone marrow cells were grown, then fixed and probed in situ with corresponding IgG controls for CD11b and F4/80 primary antibodies and subsequent fluorescent secondary antibodies. These IgG control cells were used to determine non-specific fluorescent background threshold.

## Data Availability

Not applicable.

## Supplementary Materials

The following are available online at https://www.mdpi.com/2076-3921/10/3/430/s1, Table S1: Primer sequences and corresponding annealing temperatures used for quantitative real time PCR, Table S2: Fold change of proteins detected in the conditioned media of bone marrow cells grown in LLCM + Asc over LLCM only.
