# Supplementary material for: Gene and Protein Expression Is Altered by Ascorbate Availability in Murine Macrophages Cultured under Tumour-Like Conditions
Source: Antioxidants (Basel). 2021 Mar 11;10(3):430. doi: 10.3390/antiox10030430 (PMC7998289; doi:10.3390/antiox10030430)
Supplement: Supplementary file 1 [file antioxidants-10-00430-s001.pdf]

Table S1. Primer sequences and corresponding annealing temperatures used for quantitative real time PCR.

Gene	3′ to 5′ Primer	5′ to 3′ Primer	Amplicon size (bp)	Tm
Arg 1	TTTTAGGGTTACGGCCGGTG	CCTCGAGGCTGTCCTTTTGA	146	58
β-actin	CTGTCGAGTCGCGTCCACCC	ACATGCCGGAGCCGTTGTCG	128	58
Cd206	GGCTGATTACGAGCAGTGGA	ATGCCAGGGTCACCTTTCAG	184	58
Il-6	GCCTTCTTGGGACTGATGCT	TGCCATTGCACAACTCTTTTCT	181	58
Il-10	GGTTGCCAAGCCTTATCGGA	GAGAAATCGATGACAGCGCC	156	58
Tgf-β1	AGCTGCGCTTGCAGAGATTA	AGCCCTGTATTCCGTCTCCT	189	58
Tnf-α	ACGTCGTAGCAAACCACCAA	ATAGCAAATCGGCTGACGGT	208	58
Vegfa	CTGCTGTAACGATGAAGCCCTG	GCTGTAGGAAGCTCATCTCTCC	119	58
Ym1	AGAAGCTCTCCAGAAGCAATCC	CAGTGTTCTTGTCTTTCAGACC	297	58

Table S2. Fold change of proteins detected in the conditioned media of bone marrow cells grown in LLCM + Asc over LLCM only. Isolated bone marrow cells were grown with LLCM or LLCM + Asc for 7 days at ambient air (21% O_2) or subjected to hypoxia on day 6 for ~18 h (1% O_2). Media from 3 independent experiments were pooled for a chemiluminescent protein array of 308 analytes. The table below shows the relative luminescent ratio of proteins detected in the LLCM + Asc over LLCM only group. Proteins that had relative luminescent signals below the threshold of 250 units were omitted from the final analysis, these are highlighted in red.

21% O_2	
**Protein**	**Fold LLCM + Asc / LLCM**
VE-cadherin	3.46
MMP-2	2.11
LRP-6	2.04
IL-1 R6 / IL-1 R rp2	1.98
Neurturin	1.74
IL-17R	1.65
Osteoporotegerin	1.54
NGF R / TNFRSF16	1.54
IFN-gamma R1	1.51
PF-4	1.49
IL-1 alpha	1.49
TCCR / WSX-1	1.48
CXCR6	1.43
LIGHT / TNFSF14	1.42
EG-VEGF / PK1	1.40
Follistatin like 1	1.38
TGF-beta RII	1.38
Osteoactivin / GPNMB	1.37
GFR alpha-4 / GDNF R alpha 4	1.36
IL-23 R	1.32
TGF-beta 2	1.32
GDF-5	1.32
IL-27	1.31
WISP-1 / CCN4	1.29
Lungkine	1.29
bFGF	1.27
Pentraxin3 / TSG-14	1.27
CCR9	1.26
IL-11	1.25
LIX	1.25
IGF-II	1.25
CCL28	1.23
Glut2	1.22
Lymphotoxin beta R / TNFRSF3	1.22
CXCL14 / BRAK	1.22
TIMP-2	1.21
IL-31	1.21
CD195	1.21
CD11b	1.20
IL-15 R alpha	1.19
Angiopoietin-like 2	1.19
MFRP	1.18

1% O_2	
**Protein**	**Fold LLCM + Asc / LLCM**
TRAIL / TNFSF10	9.43
IGFBP-7 / IGFBP-rp1	3.27
Dtk	3.16
MMP-2	2.85
TIMP-1	2.29
Granzyme D	1.97
IL-13	1.82
NGF R / TNFRSF16	1.81
IL-17E	1.80
MCP-1	1.74
IGFBP-5	1.73
FCrRIIB / CD32b	1.64
IGFBP-6	1.58
IL-1 RII	1.57
TROY	1.45
Osteoporotegerin	1.45
Neurturin	1.44
Urokinase	1.38
TNR RI / TNFRSF1A	1.36
ICAM-2 / CD102	1.34
Thrombospondin	1.34
uPAR	1.33
Thymus chemokine-1	1.30
SDF-1	1.29
6kine	1.27
VEGF	1.26
IL-22 BP	1.25
MCP-5	1.23
GFR alpha-3 / GDNF R alpha 3	1.23
Osteopontin	1.19
IGFBP-2	1.18
Activin A	1.18
MMP-9	1.18
IL-17D	1.17
GDF-8	1.16
P-selectin	1.16
TGF-beta 3	1.16
Galectin-3	1.15
CTLA-4 / CD152	1.15
CCR6	1.14
Leptin (OB)	1.14
MMP-3	1.12

M-CSF	1.17
Frizzled-6	1.15
Prolactin	1.15
IL-28 / IFN-lambda	1.15
IGFBP-1	1.15
P-selectin	1.14
FGF R5 beta	1.14
Endocan	1.13
IL-1 RI	1.13
IGFBP-5	1.11
TNF-beta / TNFSF1B	1.10
Granzyme B	1.10
TCA-3	1.10
VEGF-B	1.09
IL-1 R4 / ST2	1.09
HGF R	1.09
ICAM-1	1.06
MIP-2	1.05
LIF	1.05
IL-12p70	1.04
Fas / TNFRSF6	1.01
TWEAK R / TNFRSF12	1.00
IL-16	1.00
6kine	1.00
CCL1 / I-309/ TCA-3	1.00
IGFBP-3	0.99
IL-3 R alpha	0.98
TIMP-1	0.95
IGFBP-2	0.95
CTACK	0.94
Soggy-1	0.93
TROY	0.93
GDF-8	0.93
IL-1 RII	0.92
IL-2 R gamma / common gamma chain	0.91
SPARC	0.90
Endostatin	0.88
MCP-5	0.88
AgRP	0.82
TGF-beta 3	0.81
DKK-4	0.81
Progranulin	0.76
ICK	0.75
L-selectin	0.73
CCL7 / MCP-3 / MARC	0.71
ICAM-2 / CD102	0.70
Osteopontin	0.68
Urokinase	0.64
IL-22 BP	0.63
FADD	0.60
MMP-3	0.57
Neuregulin-3 / NRG3	0.57

ICAM-1	1.11
Follistatin like 1	1.11
PIGF-2	1.11
M-CSF	1.10
L-selectin	1.10
VCAM-1	1.10
Neuregulin-3 / NRG3	1.10
Endostatin	1.09
Prolactin	1.09
IL-6 R	1.09
AgRP	1.09
GFR alpha-4 / GDNF R alpha 4	1.08
TFPI	1.08
TWEAK R / TNFRSF12	1.08
IL-4	1.08
CTACK	1.07
Glut2	1.07
Frizzled-6	1.07
Pentraxin3 / TSG-14	1.06
IL-31	1.05
ICK	1.04
IL-16	1.04
PDGF R beta	1.04
IL-2 R gamma / common gamma chain	1.04
Soggy-1	1.04
IL-23	1.04
IGFBP-1	1.03
FGF R5 beta	1.03
CCL1 / I-309/ TCA-3	1.03
TCA-3	1.03
IL-15 R alpha	1.02
CCL28	1.02
LIF	1.01
Lungkine	1.01
CD11b	1.01
Fas / TNFRSF6	0.99
MFG-E8	0.99
TNF-beta / TNFSF1B	0.99
KC	0.99
MMP-24 / MT5-MMP	0.99
IL-3 R alpha	0.99
Endocan	0.99
IL-1 RI	0.98
TGF-beta RII	0.98
MIP-1 gamma	0.98
Lymphotoxin beta R / TNFRSF3	0.97
LIX	0.97
Osteoactivin / GPNMB	0.96
IL-17R	0.96
IL-1 alpha	0.96
SPARC	0.96
Epigen	0.95

VEGF	0.57
DKK-1	0.56
VCAM-1	0.52
MIP-1 gamma	0.49
TFPI	0.49
IL-13	0.46
FGF R4	0.44
PIGF-2	0.42
GFR alpha-3 / GDNF R alpha 3	0.40
CTLA-4 / CD152	0.33
SDF-1	0.33
Thrombospondin	0.32
DR3 / TNFRSF25	0.30
Activin A	0.28
KC	0.27
MMP-12	0.24
IL-10	0.23
IGFBP-7 / IGFBP-rp1	0.23
CCR6	0.22
Dtk	0.22
TNR RI / TNFRSF1A	0.20
Thymus chemokine-1	0.20
uPAR	0.20
GFR alpha-2 / GDNF R alpha 2	0.20
Galectin-3	0.20
CCL8 / MCP-2	0.19
MMP-24 / MT5-MMP	0.19
Epigen	0.19
CCR3	0.17
Epiregulin	0.15
Amphiregulin (AR)	0.11
MMP-14 / LEM-2	0.10
CXCL16	0.10
IL-5 R alpha	0.08
TLR4	0.07
MCP-1	0.05
Granzyme D	0.05
FGF R3	0.03
Insulin	N/A
TLR3	N/A
TL1A / TNFSF15	N/A
TSLP	N/A
IL-2 R alpha	N/A
S100A10	N/A
MIP-3 beta	N/A
Resistin	N/A
E selectin	N/A
SCF	N/A
Lefty-1	N/A
IGF-1	N/A
IL-17BR	N/A
Shh-N	N/A
PDGF R alpha	N/A

IL-28 / IFN-lambda	0.95
VE-cadherin	0.95
IL-1 R4 / ST2	0.95
CXCL14 / BRAK	0.95
PF-4	0.94
VEGF-B	0.94
CCL8 / MCP-2	0.93
IGFBP-3	0.93
DR3 / TNFRSF25	0.93
GFR alpha-2 / GDNF R alpha 2	0.92
MFRP	0.92
MIP-2	0.91
IL-27	0.91
Epiregulin	0.91
LRP-6	0.91
DKK-1	0.91
IL-11	0.90
IL-12p70	0.89
IL-23 R	0.88
ICAM-5	0.88
CXCR6	0.88
IFN-gamma R1	0.87
TIMP-2	0.87
MMP-12	0.87
CCL7 / MCP-3 / MARC	0.85
CCR9	0.85
LIGHT / TNFSF14	0.85
Progranulin	0.83
Amphiregulin (AR)	0.82
TCCR / WSX-1	0.82
TGF-beta 2	0.81
CXCL16	0.81
DAN	0.81
FADD	0.78
EG-VEGF / PK1	0.77
SLPI	0.76
TLR4	0.76
IGF-II	0.76
IL-24	0.75
CCR3	0.74
CD195	0.74
FGF R4	0.74
Activin RIB / ALK-4	0.73
GDF-1	0.72
GDF-3	0.71
MMP-14 / LEM-2	0.66
CCR10	0.65
Granzyme B	0.65
IFN-alpha / beta R1	0.63
HGF R	0.62
IL-1 R6 / IL-1 R rp2	0.62
GDF-5	0.61
Angiopoietin-like 2	0.61

Serum amyloid A1	N/A
IL-17RD	N/A
Granzyme G	N/A
IL-9 R	N/A
CD18 / Integrin beta 2	N/A
CD40	0.20
TRAIL / TNFSF10	0.01
Crossveinless-2	0.00
IFN-gamma	0.00
Kremen-2	0.00
TLR2	0.01
SIGIRR	0.01
TNF RII	0.01
CXCR4	0.02
Tie-2	0.02
IL-2 R alpha	0.06
MIP-3 alpha	0.05
VEGF-D	0.04
ubiquitin	0.04
IL-17C	0.02
IGFBP-6	0.01
Flt3 Ligand	0.03
ALCAM	0.02
Eotaxin-2	0.02
I-TAC	0.09
IL-20	0.04
Adiponectin / Acrp30	0.04
VEGF-C	0.04
RAGE	0.07
FCrRIIB / CD32b	0.05
Angiopoietin-like 3	0.13
IL-3	0.10
G-CSF	0.05
TREM-1	0.09
IL-17	0.07
SCF R / c-kit	0.14
HGF R	0.09
Leptin R	0.14
Fas Ligand	0.08
TRAIL R2 / TNFRSF10B	0.34
GITR Ligand / TNFSF18	0.38
GITR	0.75
CD30	0.29
TGF-beta 1	0.29
CCR7	0.17
IL-10 R alpha	0.42
MAdCAM-1	0.23
BCMA / TNFRSF17	0.17
IL-1 R9	0.42
Gremlin	0.15
BTC (betacellulin)	0.14
IL-22	0.43
IL-21 R	0.26
Fractalkine	0.21
Frizzled-1	0.29

WISP-1 / CCN4	0.59
IL-5	0.55
IL-5 R alpha	0.52
Fas Ligand	0.48
BAFF R / TNFRSF13C	0.45
DKK-4	0.42
IL-6	0.42
Decorin	0.41
bFGF	0.40
IL-2	0.24
CCR4	0.15
IL-21	98.00
IL-20	2.48
TL1A / TNFSF15	7.53
VEGF R2	8.19
TLR1	0.50
IL-7	1.29
SCF	0.69
TRAIL R2 / TNFRSF10B	2.25
IFN-beta	1.67
Insulin	N/A
TGF-beta 1	0.85
PDGF R alpha	1.36
RAGE	0.57
VEGF R3	1.86
Tie-2	0.88
MAdCAM-1	0.35
blank	0.41
TRANCE / TNFSF11	2.33
S100A10	0.01
TPO	2.47
OX40 Ligand / TNFSF4	1.23
IL-22	0.24
CD18 / Integrin beta 2	0.94
Lymphotactin	1.26
CXCR2 / IL-8 RB	0.63
VEGF-D	0.15
Gremlin	0.15
IL-2 R alpha	0.64
IFN-gamma	1.10
TREM-1	1.55
IL-21 R	0.58
VEGF-R1	1.99
IL-12 R beta 1	1.49
TSLP	1.01
Resistin	0.13
DKK-3	0.08
IL-20 R alpha	1.50
IL-31 RA	0.33
Chordin-like 2	0.49
WIF-1	N/A
TLR3	0.95
TECK	0.60
IFN-alpha / beta R2	0.29
CXCR3	0.20

TWEAK / TNFSF12	0.22
HVEM / TNFRSF14	0.37
IL-21	1.49
Chordin-like 2	0.25
CD30 L	0.37
IL-12 R beta 1	0.42
DKK-3	0.27
Tissue factor / coagulation factor III	0.29
TRANCE / TNFSF11	0.39
Growth Hormone R	0.23
Spinesin Ectodomain	0.80
IL-13 R alpha2	0.36
RANTES	0.26
CRG-2	0.43
DPPIV / CD26	0.43
EGF R	0.37
CD27 Ligand TNFSF7	0.17
Kremen-1	0.25
GDF-9	0.28
Cryptic	0.48
IL-7	0.63
TACI / TNFRSF13B	0.32
VEGF R2	0.79
beta Catenin	0.58
EDAR	0.58
B7-1/CD80	0.36
IL-6 R	0.28
CXCR2 / IL-8 RB	0.87
MDC	0.45
CD 40 Ligand / TNFSF5	0.41
TMEFF1 / Tomoregulin-1	0.23
BAFF R / TNFRSF13C	0.36
VEGF R3	0.27
Lymphotactin	0.49
CCR4	0.21
IFN-alpha / beta R2	0.34
TLR1	2.15
Activin C	0.30
IL-2	0.39
TPO	0.58
TGF-beta RI / ALK-5	0.79
Follistatin (FLRG)	0.45
Frizzled-7	1.22
TARC	0.39
CCR10	0.28
CD27 /TNFRSF7	0.46
MIP-1 alpha	0.39
FGF-21	0.62
WIF-1	0.55
PDGF C	0.47
Cerberus 1	0.31
Cardiotrophin-1	0.45
IL-3 R beta	0.49

IL-10 R alpha	0.27
MIP-1 alpha	1.12
I-TAC	0.24
Angiopoietin-like 3	0.95
Serum amyloid A1	1.14
DPPIV / CD26	0.04
TARC	0.80
EDAR	0.19
IL-4 R	0.42
IL-17RD	0.29
IL-13 R alpha2	1.57
IL-17C	1.56
TGF-beta RI / ALK-5	0.54
B7-1/CD80	0.25
IL-17	0.23
CD30 L	0.50
ubiquitin	0.76
beta Catenin	0.98
IL-3	0.54
Tissue factor / coagulation factor III	0.71
ALCAM	0.56
EGF R	0.56
CCR7	N/A
HGF R	N/A
CCL4 / MIP-1 beta	1.30
GITR	N/A
FAM3B	0.32
VEGF-C	0.93
RANTES	1.68
TWEAK / TNFSF12	0.78
CD27 /TNFRSF7	0.44
IL-7 R alpha	1.00
Shh-N	0.57
Frizzled-7	0.41
MDC	0.50
BCMA / TNFRSF17	0.19
E selectin	0.02
Adiponectin / Acrp30	0.27
Cardiotrophin-1	0.52
Endoglin / CD105	0.84
MIP-3 beta	0.20
IL-17BR	0.66
Granzyme G	0.79
MIP-3 alpha	0.43
IL-9 R	0.54
PDGF C	0.62
IL-17RC	0.39
SCF R / c-kit	0.35
Eotaxin	0.81
CRG-2	0.79
HVEM / TNFRSF14	0.60
GM-CSF	0.81
Csk	0.83

CXCR3	0.73		IL-1 R9	0.54
PDGF R beta	0.40		GITR Ligand / TNFSF18	0.61
IFN-beta	0.68		IL-18 R alpha / IL-1 R5	0.56
TNF-alpha	0.38		TIMP-4	0.91
IL-24	0.69		CD14	0.37
MMP-9	0.40		Kremen-1	0.28
DAN	0.45		Erythropoeitin (EPO)	N/A
BLC	0.36		Follistatin (FLRG)	0.10
Endoglin / CD105	3.48		IL-17F	0.01
Artemin	0.42		TLR2	0.92
IL-31 RA	2.98		RELM beta	1.13
IL-9	0.72		CXCR4	0.09
Erythropoeitin (EPO)	N/A		TNF RII	0.68
IL-17RC	1.00		Flt3 Ligand	0.53
IL-17E	0.70		FGF-21	0.36
Eotaxin	0.79		NOV / CCN3	0.52
OX40 Ligand / TNFSF4	1.16		IL-15	0.93
IL-4	0.44		Crossveinless-2	0.15
IL-1 beta	0.88		TACI / TNFRSF13B	0.65
Decorin	0.72		IL-3 R beta	1.01
Axl	0.68		BTC (betacellulin)	0.66
TSLP R	1.11		Artemin	1.01
IL-4 R	1.28		FGF R3	0.67
Csk	0.95		Frizzled-1	0.69
NOV / CCN3	1.78		IL-12p40/p70	0.80
MFG-E8	0.89		IL-10	0.84
IL-23	0.84		Leptin R	0.68
IL-12p40/p70	0.75		CD40	0.20
TIMP-4	1.51		IL-2 R alpha	0.09
CD14	0.70		CD 40 Ligand / TNFSF5	0.34
Leptin (OB)	0.61		CD27 Ligand TNFSF7	0.45
SLPI	0.68		IGF-1	0.25
MIG	1.03		TNF-alpha	0.91
GDF-3	0.74		Axl	0.37
IL-15	1.24		Spinesin Ectodomain	0.54
ICAM-5	0.64		CD30	0.55
IL-7 R alpha	1.02		Growth Hormone R	0.21
RELM beta	0.97		Fractalkine	0.67
IFN-alpha / beta R1	1.05		GDF-9	0.97
blank	0.81		G-CSF	0.77
FAM3B	2.14		IL-1 beta	0.53
IL-6	0.86		Activin C	0.26
IL-18 R alpha / IL-1 R5	0.88		SIGIRR	0.30
IL-20 R alpha	1.21		Lefty-1	N/A
GDF-1	0.89		Kremen-2	0.64
IL-17D	0.67		Cryptic	0.29
VEGF-R1	1.39		TSLP R	1.00
GM-CSF	7.66		IL-9	0.56
CCL4 / MIP-1 beta	1.60		TMEFF1 / Tomoregulin-1	0.91
IL-17F	1.59		MIG	0.81
IL-5	0.88		Eotaxin-2	0.74
Activin RIB / ALK-4	0.85		BLC	0.66
TECK	1.61		Cerberus 1	0.87
